# The Effect of Repeated abobotulinumtoxinA (Dysport®) Injections on Walking Velocity in Persons with Spastic Hemiparesis Caused by Stroke or Traumatic Brain Injury

**DOI:** 10.1002/pmrj.12459

**Published:** 2020-09-11

**Authors:** Alberto Esquenazi, Allison Brashear, Thierry Deltombe, Monika Rudzinska‐Bar, Malgorzata Krawczyk, Alexander Skoromets, Michael W. O'Dell, Anne‐Sophie Grandoulier, Claire Vilain, Philippe Picaut, Jean‐Michel Gracies

**Affiliations:** ^1^ MossRehab Elkins Park PA USA; ^2^ University of California Davis CA USA; ^3^ Service de Médecine Physique et Réadaptation, Centre Hospitalier Universitaire UCL Yvoir Belgium; ^4^ Department of Neurology, Faculty of Medicine and Health Service Andrzej Frycz Modrzewski Krakow University Krakow Poland; ^5^ Centrum Medyczne Plejady ul Kraków Poland; ^6^ Pavlov First St Petersburg State Medical University St Petersburg Russia; ^7^ Department of Rehabilitation Medicine Weill Cornell Medicine New York NY USA; ^8^ Ipsen Innovation Les Ulis France; ^9^ EA 7377 BIOTN, Université Paris‐Est Créteil, Service de Rééducation Neurolocomotrice, Hôpitaux Universitaires Henri Mondor Créteil France

## Abstract

**Background:**

Botulinum toxin (BoNT) injections were shown to improve muscle tone of limbs in patients with spasticity. However, limited data are available regarding the effects of repeated BoNT injections on walking ability.

**Objective:**

To assess changes in walking velocity (WV), step length, and cadence under different test conditions after repeated treatment with abobotulinumtoxinA (aboBoNT‐A; Dysport) in spastic lower limb muscles.

**Design:**

Secondary analysis of an open‐label, multiple‐cycle extension (National Clinical Trials number NCT01251367) to a phase III, double‐blind, randomized, placebo‐controlled, single‐treatment cycle study, in adults with chronic hemiparesis (NCT01249404).

**Setting:**

Fifty‐two centers across Australia, Belgium, the Czech Republic, France, Hungary, Italy, Poland, Portugal, Russia, Slovakia, and the United States.

**Patients:**

352 Ambulatory adults (18‐80 years) with spastic hemiparesis and gait dysfunction caused by stroke or traumatic brain injury, with a comfortable barefoot WV of 0.1 to 0.8 m/s.

**Interventions:**

Up to four aboBoNT‐A treatment cycles, administered to spastic lower limb muscles.

**Main Outcome Measurements:**

Changes from baseline in comfortable and maximal barefoot and with shoes WV (m/s), step length (m/step), and cadence (steps/minutes).

**Results:**

At Week 12 after four injections, WV improved by 0.08 to 0.10 m/s, step length by 0.03 to 0.04 m/step, and cadence by 3.9 to 6.2 steps/minutes depending on test condition (all *P* < .0001 to .0003 vs baseline). More patients (7% to 17%) became unlimited community ambulators (WV ≥0.8 m/s) across test conditions compared with baseline, with 39% of 151 patients classified as unlimited community ambulators in at least one test condition and 17% in all four test conditions.

**Conclusions:**

Clinically meaningful and statistically significant improvements in WV, step length, and cadence under all four test conditions were observed in patients with spastic hemiparesis after each aboBoNT‐A treatment cycle.

## Introduction

Walking velocity (WV) in patients with chronic hemiparesis following stroke or traumatic brain injury is typically limited, stabilizing at a level that leaves many patients unable to functionally ambulate in the community.^1^, ^2^ Limitations in walking ability can lead to a decline in participation in a normal life and may accelerate a decline in health.^3^, ^4^ Improvements in the ability to walk in the community are therefore a desirable primary goal for many patients with hemiparesis.

Gait velocity is used as an outcome to assess community ambulation potential in patients who have experienced a stroke.^3^ Patients with a WV of at least 0.8 m/s are considered to have unlimited community ambulatory capacity, whereas those with a WV of 0.4 to 0.8 m/s are considered limited community ambulators and those with a WV of less than 0.4 m/s are considered household ambulators.^3^

Spastic hemiparesis may contribute to gait dysfunction as a result of impaired motor control, abnormal muscle activation, muscle weakness, and muscle extensibility loss.^5^, ^6^ Botulinum neurotoxin type A (BoNT‐A) is approved, recommended, and used as a treatment for overactive muscles in spastic paresis.^7^, ^8^ AbobotulinumtoxinA (aboBoNT‐A, Dysport [Ipsen]) has been shown to reduce muscle tone and improve subjective functional outcomes in both the upper and lower limbs.^9^, ^10^, ^11^, ^12^ Recently published data from a multicenter, prospective, double‐blind, randomized, placebo‐controlled, single‐cycle, adult lower limb study, followed by a 1‐year open‐label, multiple‐cycle extension study, have shown that aboBoNT‐A 1500 U significantly reduced muscle tone (assessed using the Modified Ashworth Scale [MAS] in the gastrocnemius–soleus complex) at Weeks 4 and 12 post injection in the double‐blind phase.^10^ In the open‐label extension, muscle tone improvements were maintained with repeated aboBoNT‐A injections, and progressive improvements were observed in physician global assessment, angle of catch (Tardieu Scale for spasticity), active range of ankle dorsiflexion, and quality of life parameters. Long‐term safety and tolerability were good.^10^

A secondary outcome of this study was 10‐m comfortable barefoot WV without walking aids, a common test for assessing gait in hemiparesis.^3^ In the adult lower limb study, the mean baseline WV was 0.45 m/s. This progressively increased with repeated injections, with a mean improvement of 25% after five injections (Week 4 of open‐label Cycle 4) relative to baseline. After four injections (open‐label Cycle 3), and a time span of >9 months (identified as chronic phase of hemiparesis) during which WV normally plateaus at ~0.7 m/s,^2^, ^13^, ^14^ 16% of patients achieved an increase in WV of at least 0.8 m/s, a threshold associated with community mobility, compared with 0% of patients at baseline.^10^ In general, larger increases in WV were observed at Week 12 compared with Week 4 across injections, as opposed to other outcome measures, such as muscle tone; this may reflect the time needed for patients to adapt to changes in muscle tone and joint range of motion in the lower limb for functional improvements in WV.^10^

Along with comfortable barefoot WV analyses, there were other assessments of WV performed in the adult lower limb study, including comfortable WV with shoes, maximal barefoot WV, and maximal WV with shoes, with change in functional ambulatory category (according to WV) also examined over treatment cycles. In addition, changes in step length and cadence were assessed to determine if the changes in WV were achieved by increasing step length, cadence, or both.^15^

Ambulation improvement provides value in terms of life participation, quality of life, and reduction of medical complications and is an important treatment goal for many patients.^1^, ^4^, ^16^ In order to further understand the functional improvements that can be achieved with aboBoNT‐A, here we report the secondary analyses from multiple walking assessments made in the adult lower limb study. The aim of this analysis was to establish the efficacy of repeated injections of the lower limb with aboBoNT‐A on improvements in WV, step length, and cadence compared with baseline in adults with spastic hemiparesis of the lower limb. These analyses are based on secondary outcomes from a phase III double‐blind and open‐label study. Here we also assess if such improvements correspond to movement between ambulatory categories for household, limited community, and unlimited community ambulation.^17^

## Methods

### Study Design

Details of the adult lower limb double‐blind (NCT01249404) and open‐label (NCT01251367) studies have been previously published.^10^ In brief, the double‐blind study was a multicenter, prospective, randomized, placebo‐controlled, single‐treatment‐cycle study in adults with chronic hemiparesis and consisted of a single injection with 1000 U or 1500 U of aboBoNT‐A or placebo to affected lower limbs. The open‐label phase was a multicenter, prospective extension that involved up to four repeat injections of 1000 U or 1500 U of aboBoNT‐A.

The double‐blind study primary objective was to demonstrate single aboBoNT‐A injection efficacy (principally, reducing lower extremity muscle tone) versus placebo in the lower extremity; safety was a secondary objective. The open‐label study primary objective was to assess long‐term safety of repeated aboBoNT‐A injections; long‐term efficacy was a secondary objective. The research protocol and all study documents were approved by an independent ethics committee and the study was conducted in accordance with the Declaration of Helsinki and the International Conference on Harmonisation Consolidated Guidelines on Good Clinical Practice.^18^, ^19^ All participants provided written informed consent prior to participation in the study.

### Treatment Administration

In each injection cycle, aboBoNT‐A injections were administered to the gastrocnemius‐soleus muscle complex, with additional injections to the tibialis posterior, flexor digitorum longus, flexor digitorum brevis, flexor hallucis longus, flexor hallucis brevis, rectus femoris, hamstrings, adductor magnus, gracilis, or gluteus maximus based on the investigator's clinical judgment. Injections could also be administered to the affected upper limb based on the investigator's clinical judgment from the beginning of open‐label Cycle 3. Patients were not required to meet the initial study inclusion criteria in order to receive injection at each cycle. The need for retreatment was considered at each study visit after Week 12 of each treatment, with optional follow‐up visits at Week 16, 20, and 24 of each cycle. Retreatment decisions were based on the following reinjection criteria: a MAS score within 1 grade of the double‐blind study baseline value, a physician's global assessment score that showed no improvement (≤0) compared with double‐blind study baseline, and no unacceptable safety risk based on investigators' clinical judgment. The maximum study duration was 18 months (double‐blind phase, 6 months; open‐label phase, 12 months), during which patients could receive a maximum of five injections (including double‐blind cycle). If patients did not require reinjection at the Week 24 visit of open‐label Cycle 1 or 2, they attended follow‐up visits every 4 weeks until they either met the retreatment criteria and entered the next treatment cycle or completed at least 12 months of follow‐up.

As per the study protocol, no standardized physiotherapy was administered and could not be initiated 4 weeks prior to study entry or during the first 4 weeks of the study.

### Participants

Ambulatory patients aged between 18 and 80 years with spastic hemiparesis causing gait dysfunction; comfortable barefoot WV between 0.1 and 0.8 m/s, measured with a 10‐m WV test (WVT) without walking aids; and only one clinically defined stroke episode^20^ or brain trauma ≥6 months prior to enrollment into the double‐blind study. Patients, including those from the placebo group, were eligible for the open‐label phase if they had participated in the double‐blind study and completed the Week 12, 16, 20, or 24 follow‐up visit, without any major protocol deviations and/or any ongoing adverse events, either of which, in the opinion of the investigator, would pose an unacceptable risk to the patients were they to continue receiving treatment in the open‐label extension study. Patients could enter the open‐label extension study at any time after Week 12.

### Assessments

Here we report a detailed statistical analysis of changes in WVTs, step length, and cadence of the patients treated with aboBoNT‐A over the first four injections (one double‐blind and the first three open‐label injections) assessed at Week 12 of each cycle relative to preinjection baseline of the double‐blind study. Some patients received five injections (open‐label Cycle 4), but the last mandatory study visit was at Week 4 of this cycle; therefore, Week 12 data were not available (n = 0). As such, the fifth injection cycle was not included for these analyses. Walking ability was assessed through measurement of comfortable barefoot WV, maximal barefoot WV, comfortable WV with shoes, and maximal WV with shoes. In all WV tests, patients were asked to walk for 10 m on the same flat floor at all visits, without obstacles or turns. Duration was measured with a stopwatch from the time the first foot crossed the starting mark to when a foot crossed the arrival mark. The evaluator walked beside the patient and measured the time and the number of steps taken during the 10 m. Space for acceleration and deceleration was provided. A 1‐minute resting period was scheduled between each of the walking tests.

Data are provided at Week 12 post injection up to the fourth injection (open‐label Cycle 3). Patients were also categorized and grouped based on Perry et al's classification for WV^17^: WV of at least 0.8 m/s (unlimited community ambulators), 0.4 to 0.8 m/s (community limited ambulators), and less than 0.4 m/s (household ambulators). The shifts between ambulatory categories after each treatment cycle and quantitative gain in reaching the unlimited community ambulation category were quantified. Results from patients who received either 1000 U or 1500 U of aboBoNT‐A are grouped as open‐label results for muscle tone improvements, physician global assessment score, and comfortable barefoot WV were similar between doses as previously reported.^10^ Some patients did not require retreatment at subsequent cycles and not all patients completed every assessment type at Week 12; all available data for each assessment and at each treatment cycle are presented.

### Statistical Analyses

Numerical differences between baseline and Week 12 post‐injection results are presented (absolute values) in the four walking test conditions, alongside percentage improvement values (mean of individual percentage change from baseline calculated for each patient). An intragroup analysis was performed after each injection to compare the Week 12 values with the initial baseline values.

In addition, a statistical model was used to analyze the change from baseline to Week 12 following each injection. Considering the context of multiple walking assessments, a mixed model for repeated measures (MMRM) has been used, including the fixed categorical effects of test condition, visit, test condition‐by‐visit interaction, test condition‐by‐baseline interaction, and baseline value as a fixed continuous covariates. Least squares (LS) mean changes from baseline as well as 95% confidence intervals (CIs) are provided at Week 12 of each cycle, under each test condition for walking velocity, step length, and cadence.

## Results

Patient demographics, patient disposition, and BoNT‐A dose administered to each muscle group across cycles have been previously reported.^10^ Of 366 patients who completed the double‐blind study, 352 were eligible for the open‐label extension. The mean age of patients was 53.2 years of age (ranging from 21‐80), 67.9% were male, and approximately 60% received concomitant physiotherapy. Nearly one third (n = 104) of patients receiving 1000 U of aboBoNT‐A in the lower limb also received 500 U in their upper limb (98 during Cycle 3; 67 during Cycle 4).

Depending on the number of injections received, mean comfortable barefoot WV (SD) at baseline ranged between 0.43 (0.20) and 0.45 (0.22) m/s. In other test conditions, baseline values were between 0.56 (0.30) and 0.60 (0.33) m/s for maximal barefoot WV; 0.48 (0.22) and 0.49 (0.23) m/s for comfortable WV with shoes; and 0.64 (0.33) and 0.66 (0.33) m/s for maximal WV with shoes (Table S1).

**Figure 1:**
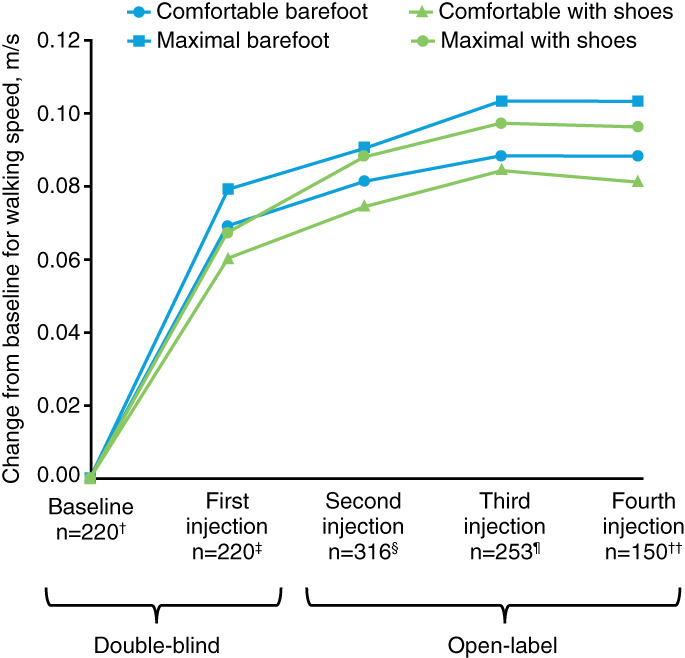
Mean change from baseline for walking velocity under four conditions at Week 12 of each cycle from the double‐blind study to the open‐label study (all doses combined). ^†^219 for maximal WV with shoes; ^‡^219 for maximal WV with shoes; ^§^317 for maximal WV barefoot, 319 for comfortable WV with shoes, 318 for maximal WV with shoes; ^¶^254 for comfortable WV with shoes and maximal WV with shoes; ^††^151 for comfortable WV with shoes and maximal WV with shoes. WV = walking velocity.

Improvements in WV were observed in the comfortable barefoot walking test across the four injections with a mean (SD) improvement from baseline to Week 12 (mean percentage improvement) of +0.09 (0.14) m/s (+23.6%) at Week 12 after the fourth injection. Improvements observed at Week 12 after each injection for comfortable barefoot WV were statistically significant compared with baseline values (all *P* < .0001; Table 1 and Figure 1). After the fourth injection, the estimated LS mean change from baseline was 0.095 m/s (95% CI: 0.071; 0.12) (Table S2).

**Table 1:** Walking pattern across injection cycles in the comfortable barefoot category at Baseline and Week 12 (aboBoNT‐A doses combined)

	Double‐blind	Open‐label		
	First injection	Second injection	Third injection	Fourth injection
**Walking Velocity (m/s)**	n = 220	n = 316	n = 253	n = 150
Baseline,^*^ Mean (SD)	0.452 (0.22)	0.448 (0.22)	0.441 (0.22)	0.433 (0.20)
Mean (SD) at W12	0.521 (0.25)	0.528 (0.26)	0.529 (0.27)	0.521 (0.27)
Mean change (SD) at W12	0.069 (0.11)	0.081 (0.13)	0.088 (0.14)	0.088 (0.14)
*P* value (change from baseline)	<.0001	<.0001	<.0001	<.0001
Percentage change from baseline at W12 (95% CI)	19.68 (14.63, 24.73)	21.59 (17.41, 25.78)	25.08 (19.80, 30.35)	23.58 (17.41, 29.76)

**Step Length (m/step)**	n = 220	n = 316	n = 253	n = 150
Baseline,^*^ Mean (SD)	0.345 (0.13)	0.350 (0.13)	0.346 (0.14)	0.347 (0.13)
Mean (SD) at W12	0.370 (0.14)	0.383 (0.14)	0.387 (0.15)	0.387 (0.16)
Mean change (SD) at W12	0.025 (0.06)	0.033 (0.07)	0.041 (0.07)	0.040 (0.08)
*P* value (change from baseline)	<.0001	<.0001	<.0001	<.0001
Percentage change from baseline at W12 (95% CI)	9.41 (6.64, 12.86)	11.77 (9.37, 14.17)	14.48 (11.44, 17.51)	13.80 (9.45, 18.15)

**Cadence (steps/min)**	n = 220	n = 316	n = 253	n = 150
Baseline,^*^ Mean (SD)	76.7 (19.8)	75.2 (20.4)	74.9 (20.5)	73.7 (20.2)
Mean (SD) at W12	82.5 (20.8)	80.3 (21.8)	79.8 (21.6)	78.7 (22.1)
Mean change (SD) at W12	5.8 (11.8)	5.1 (12.7)	4.9 (13.3)	5.0 (12.3)
*P* value (change from baseline)	<.0001	<.0001	<.0001	<.0001
Percentage change from baseline at W12 (95% CI)	9.71 (6.21, 13.21)	8.39 (6.02, 10.76)	8.66 (5.89, 11.43)	8.43 (5.23, 11.62)

^*^ Baseline refers to baseline of the double‐blind study, prior to first injection of the patients entering the cycle. aboBoNT‐A, abobotulinumtoxinA; CI, confidence interval; SD, standard deviation; W12, Week 12.

Similar improvements in WV were identified across the three other walking test conditions, which were also statistically significant compared with baseline at Week 12 after each injection (all *P* < .0001; Table S1 and Figure 1). Mean (SD) improvements from baseline to Week 12 (mean percentage improvement) after the fourth injection (open‐label Cycle 3) were + 0.10 (0.19) m/s (+22.9%) for maximal barefoot WV, +0.08 (0.15) m/s (+22.6%) for comfortable WV with shoes, and + 0.10 (0.20) m/s (+19.7%) for maximal WV with shoes. The corresponding estimated LS mean changes from baseline can be found in Table S2.

Among patients tested at comfortable WV who received four injections, 47% were classified as household ambulators (WV <0.4 m/s) and 53% as limited ambulators (WV 0.4 to 0.8 m/s) at baseline (Table S3). After four injections, 17% of these patients were classified as unlimited community ambulators (≥0.8 m/s; compared with 0% at baseline), achieving a mean (SD) quantitative gain of 0.27 (0.15) m/s (minimum, +0.04 m/s; maximum, +0.56 m/s) in order to reach this ambulatory category (Table S4). Increases in the proportion of patients with improving levels of community ambulatory capacity following the fourth injection were also observed in the other three test conditions, with 21% to 38% of patients considered unlimited community ambulators across these conditions, although the differences versus baseline were less marked (7% to 12%) (Table S3).

Globally, 39% (n = 59/151) of the patients who had received four injections achieved unlimited community ambulation in at least one test condition, 31% (n = 47/151) in two or more conditions, 22% (n = 33/151) in three or more conditions, and 17% (n = 26/151) in all four conditions.

**Figure 2:**
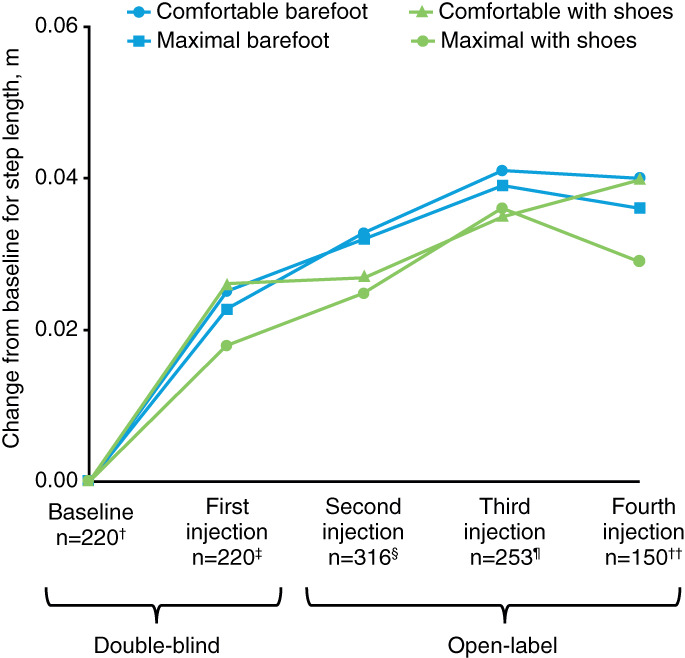
Mean change from baseline for step length under four conditions at Week 12 of each cycle from the double‐blind study to the open‐label study (all doses combined). ^†^219 for maximal with shoes; ^‡^219 for maximal with shoes; ^§^317 for maximal barefoot, 319 for comfortable with shoes, 318 for maximal with shoes; ^¶^254 for comfortable with shoes and maximal with shoes; ^††^151 for comfortable with shoes and maximal with shoes.

**Figure 3:**
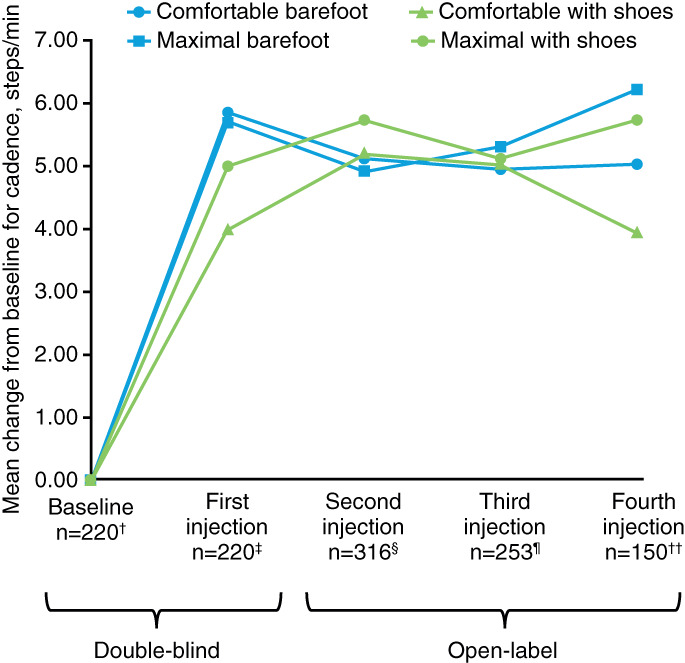
Mean change from baseline for cadence under four conditions at Week 12 of each cycle from the double‐blind study to the open‐label study (all doses combined). ^†^219 for maximal with shoes; ^‡^219 for maximal with shoes; ^§^317 for maximal barefoot, 319 for comfortable with shoes, 318 for maximal with shoes; ^¶^254 for comfortable with shoes and maximal with shoes; ^††^151 for comfortable with shoes and maximal with shoes.

Increases in WV can be achieved by increasing step length, cadence, or both; in the present study, patients appeared to achieve improvements through both. Improvements in step length and cadence were observed to a similar extent across each of the four walking test conditions by the fourth injection. For comfortable barefoot WV, step length improved by a mean (SD; mean percentage improvement) of +0.04 (0.08) m/step (+13.8%) with an improvement in cadence of +5.0 (12.3) steps/minutes (+8.4%) at Week 12 after the fourth injection (Table 1 and Figures 2 and 3). According to the intragroup, improvements observed at Week 12 after each injection for comfortable barefoot WV were statistically significant compared with baseline values (all *P* < .0001). Similar statistically significant improvements were also seen across the three other walking test conditions (*P* < .0001 to *P* = .0003; Table S1, Figures 2 and 3): at maximal barefoot WV, there was a mean (SD) improvement (mean percentage improvement) of +0.04 (0.09) m/step (+11.2%) in step length with an improvement in cadence of +6.2 (15.2) steps/minutes (+9.7%); at comfortable WV with shoes, a mean (SD) improvement (mean percentage improvement) of +0.04 (0.09) m/step (+13.2%) in step length and + 3.9 (12.6) steps/minutes (+7.2%) in cadence was seen; and at maximal WV with shoes, a mean (SD) improvement (mean percentage improvement) of +0.03 (0.10) m/step (+9.5%) in step length and + 5.7 (15.7) steps/minutes (+8.6%) in cadence was observed. The corresponding estimated LS mean changes from baseline can be found in Table S2.

## Discussion

In this secondary analysis, repeated aboBoNT‐A treatment cycles were associated with sustained improvements in walking ability in patients with chronic spastic hemiparesis. Improvements observed at Week 12 post injection were statistically significant compared with baseline values at all cycles and for each walking test condition.

WV normally plateaus at approximately 0.7 m/s in chronic (>9 months) hemiparesis.^2^, ^13^, ^14^ Following the fourth injection in the present study (and therefore >9 months since double‐blind baseline; n = 151), 39% of patients were walking at ≥0.8 m/s, a threshold associated with unlimited community mobility,^3^ at least for one test condition (compared with 0% at baseline), and 17% under all four test conditions. This move between ambulatory categories highlights the clinical relevance of the observed improvements in WV. Across all four test conditions, WV increased from baseline by between 0.08 and 0.10 m/s (20% and 24%) after four injections. These improvements were also achieved in parallel with increases in physician‐rated global assessment and patient‐rated quality of life, as reported in the primary publication.^10^

Step length improved by 0.03 to 0.04 m/step (10% to 14%) across all test conditions after four injections, and cadence improved by 3.9 to 6.2 steps/minutes (7% to 10%). Along with a mean improvement in comfortable barefoot WV of around 0.09 m/s (20%) and improvement in ambulatory capacity, this represents a clinically meaningful achievement in chronic hemiparesis.^16^ Using MMRM analysis, as shown in Supplemental Table 2, a similar pattern of improvements was observed across WV for all test conditions, as did step length and cadence, although in each case LS mean change from baseline values were slightly increased compared with the absolute mean change values.

Other studies have reported improvements in walking ability following BoNT‐A injections of the lower limb using a number of assessment techniques, including timed walking tests, Fugl‐Meyer assessments, use of video technology to measure temporospatial parameters, or instrumented insoles,^8^, ^21^, ^22^, ^23^, ^24^ with greater improvements in one study when patients underwent an adjunct guided self‐rehabilitation program.^25^

In the primary study, comfortable barefoot WV improvement was consistently greater at Week 12 compared with Week 4,^10^ which contrasts with other outcome measures and prior placebo‐controlled botulinum toxin studies.^26^, ^27^ It could be that, in order to fully take advantage of the newfound gains, an accommodation period is needed while patients adapt their walking pattern to a new physiological baseline, with reduced muscle co‐contraction and increased joint range of motion produced by aboBoNT‐A.^25^ Furthermore, walking improvements may also be explained by brain plasticity processes as a result of improved lower limb function after repeated treatment cycles. Overall, these results suggest that optimal post‐injection walking assessment times in future studies may be at 12 weeks.

Patients with hemiparesis have previously reported improved quality of life with increased WV following repeated aboBoNT‐A treatments but not after a single BoNT‐A treatment.^10^, ^28^ Increased independence for ambulation and daily activities affects quality of life and mental health for patients and reduces burden on caregivers.^29^, ^30^ Increases in WV may result in gains in overall fitness and a reduction in comorbidities related to immobility, such as cardiovascular disease, depression, and the need for institutionalization, with a reduction in healthcare needs.^4^, ^31^, ^32^ The results of this study are important for the chronic stroke population; moving patients across the established ambulatory categories^17^ is not easy to achieve, but it is of great importance and clinical relevance.^16^

## Limitations

Patients with a comfortable barefoot WV of more than 0.8 m/s at baseline were excluded from the study, which represents a potential study bias. Additionally, improvements in walking velocity were not the primary endpoint of the studies from which these data were obtained, and analyses by ambulatory category, for the MMRM and statistical analyses of changes from baseline were not planned, but post hoc analyses.

## Conclusions

Repeated aboBoNT‐A injections to the lower limb were associated with sustained and statistically significant improvements in WV, step length, and cadence in patients with chronic spastic hemiparesis after stroke or traumatic brain injury. Improvements were progressive and greater after several treatment cycles. These improvements in WV were also shown to have a positive impact on community ambulation achievement in some patients.

### Data Statement

Where patient data can be anonymized, Ipsen will share all individual participant data that underlie the results reported in this article with qualified researchers who provide a valid research question. Study documents, such as the study protocol and clinical study report, are not always available. Proposals should be submitted to datasharing@ipsen.com and will be assessed by a scientific review board. Data are available beginning 6 months and ending 5 years after publication; after this time, only raw data may be available.

## Supporting information


**Appendix S1** Supporting Information
