# Supplementary material for: The Effect of Repeated abobotulinumtoxinA (Dysport®) Injections on Walking Velocity in Persons with Spastic Hemiparesis Caused by Stroke or Traumatic Brain Injury
Source: PM R. 2020 Sep 11;13(5):488–95. doi: 10.1002/pmrj.12459 (PMC8246752; doi:10.1002/pmrj.12459)
Supplement: Supplementary file 1 — Appendix S1 Supporting Information [file PMRJ-13-488-s001.docx]

**Supplementary**

**Repeated abobotulinumtoxinA (Dysport^®^) injection effect on walking velocity in persons with spastic hemiparesis caused by stroke or traumatic brain injury**

**Supplementary Table 1** Walking pattern across injection cycles in each of the four walking test conditions at Baseline and Week 12 (aboBoNT-A doses combined)

|  | **Double-blind** | | **Open-label** | | | | | |
| --- | --- | --- | --- | --- | --- | --- | --- | --- |
| **WALKING VELOCITY (m/s)** | **First injection** | | **Second injection** | | **Third injection** | | **Fourth injection** | |
| **Comfortable barefoot walking velocity** | | | | | | | | |
| Baseline,^†^ Mean (SD)  Mean (SD) at W12  Mean change (SD) at W12  P-value (change from baseline)  Percentage change from baseline at W12 (95% CI) | n=220  0.452 (0.22)  0.521 (0.25)  0.069 (0.11)  <0.0001  19.68 (14.63, 24.73) | | n=316  0.448 (0.22)  0.528 (0.26)  0.081 (0.13)  <0.0001  21.59 (17.41, 25.78) | | n=253  0.441 (0.22)  0.529 (0.27)  0.088 (0.14)  <0.0001  25.08 (19.80, 30.35) | | n=150  0.433 (0.20)  0.521 (0.27)  0.088 (0.14)  <0.0001  23.58 (17.41, 29.76) | |
| **Maximum barefoot walking velocity** | | | | | | | | |
| Baseline,^†^ Mean (SD)  Mean (SD) at W12  Mean change (SD) at W12  P-value (change from baseline)  Percentage change from baseline at W12 (95% CI) | n=220  0.596 (0.33)  0.675 (0.36)  0.079 (0.14)  <0.0001  18.58 (13.30, 23.85) | | n=317  0.583 (0.32)  0.673 (0.37)  0.090 (0.16)  <0.0001  20.09 (15.74, 24.44) | | n=253  0.579 (0.31)  0.681 (0.38)  0.103 (0.17)  <0.0001  23.68 (17.92, 29.44) | | n=150  0.564 (0.30)  0.667 (0.38)  0.103 (0.19)  <0.0001  22.86 (15.75, 29.97) | |
| **Comfortable walking velocity with shoes** | | | | | | | | |
| Baseline,^†^ Mean (SD)  Mean (SD) at W12  Mean change (SD) at W12  P-value (change from baseline)  Percentage change from baseline at W12 (95% CI) | n=220  0.492 (0.23)  0.551 (0.24)  0.060 (0.11)  <0.0001  17.82 (12.81, 22.83) | | n=319  0.487 (0.23)  0.561 (0.26)  0.074 (0.13)  <0.0001  20.66 (15.73, 25.59) | | n=254  0.485 (0.23)  0.569 (0.26)  0.084 (0.14)  <0.0001  24.27 (18.39, 30.16) | | n=151  0.482 (0.22)  0.563 (0.27)  0.081 (0.15)  <0.0001  22.63 (14.67, 30.59) | |
| **Maximum walking velocity with shoes** | | | | | | | | |
| Baseline,^†^ Mean (SD)  Mean (SD) at W12  Mean change (SD) at W12  P-value (change from baseline)  Percentage change from baseline at W12 (95% CI) | n=219  0.660 (0.33)  0.728 (0.35)  0.068 (0.14)  <0.0001  14.47 (9.86, 19.07) | | n=318  0.649 (0.33)  0.737 (0.38)  0.088 (0.17)  <0.0001  17.72 (13.22, 22.22) | | n=254  0.648 (0.33)  0.746 (0.38)  0.097 (0.18)  <0.0001  20.67 (14.94, 26.41) | | n=151  0.641 (0.33)  0.737 (0.39)  0.096 (0.20)  <0.0001  19.73 (12.42, 27.05 | |
| **STEP LENGTH [m/step]** |  |  | |  | |  | |  |
| **Comfortable barefoot walking velocity** | | | | | | | | |
| Baseline,^†^ Mean (SD)  Mean (SD) at W12  Mean change (SD) at W12  P-value (change from baseline)  Percentage change from baseline W12 (95% CI) | n=220  0.345 (0.13)  0.370 (0.14)  0.025 (0.06)  <0.0001  9.41 (6.64, 12.86) | | n=316  0.350 (0.13)  0.383 (0.14)  0.033 (0.07)  <0.0001  11.77 (9.37, 14.17) | | n=253  0.346 (0.14)  0.387 (0.15)  0.041 (0.07)  <0.0001  14.48 (11.44, 17.51) | | n=150  0.347 (0.13)  0.387 (0.16)  0.040 (0.08)  <0.0001  13.80 (9.45, 18.15) | |
| **Maximum barefoot walking velocity** | | | | | | | | |
| Baseline,^†^ Mean (SD)  Mean (SD) at W12  Mean change (SD) at W12  P-value (change from baseline)  Percentage change from baseline at W12 (95% CI) | n=220  0.389 (0.15)  0.412 (0.15)  0.023 (0.07)  <0.0001  8.29 (5.48, 11.11) | | n=317  0.392 (0.16)  0.425 (0.16)  0.032 (0.08)  <0.0001  10.87 (8.49, 13.25) | | n=253  0.391 (0.16)  0.430 (0.17)  0.039 (0.09)  <0.0001  13.22 (10.03, 16.41) | | n=150  0.390 (0.15)  0.426 (0.17)  0.036 (0.09)  <0.0001  11.22 (7.04, 15.40) | |
| **Comfortable walking velocity with shoes** | | | | | | | | |
| Baseline,^†^ Mean (SD)  Mean (SD) at W12  Mean change (SD) at W12  P-value (change from baseline)  Percentage change from baseline at W12 (95% CI) | n=220  0.374 (0.13)  0.400 (0.14)  0.026 (0.07)  <0.0001  9.28 (6.21, 12.37) | | n=319  0.380 (0.14)  0.407 (0.14)  0.028 (0.07)  <0.0001  9.93 (7.35, 12.53) | | n=254  0.377 (0.14)  0.413 (0.14)  0.035 (0.08)  <0.0001  13.01 (9.73, 16.28) | | n=151  0.379 (0.13)  0.419 (0.16)  0.040 (0.09)  <0.0001  13.24 (8.68, 17.79) | |
| **Maximum walking velocity with shoes** | | | | | | | | |
| Baseline,^†^ Mean (SD)  Mean (SD) at W12  Mean change (SD) at W12  P-value (change from baseline)  Percentage change from baseline at W12 (95% CI) | n=219  0.433 (0.15)  0.451 (0.16)  0.018 (0.07)  0.0002  6.09 (3.59, 8.59) | | n=318  0.436 (0.16)  0.460 (0.16)  0.025 (0.08)  <0.0001  7.97 (5.72, 10.23) | | n=254  0.433 (0.16)  0.469 (0.17)  0.036 (0.09)  <0.0001  11.50 (8.26, 14.73) | | n=151  0.436 (0.16)  0.465 (0.17)  0.029 (0.10)  0.0003  9.46 (5.12, 13.80) | |
| **CADENCE [steps/min]** |  |  | |  | |  | |  |
| **Comfortable barefoot walking velocity** | | | | | | | | |
| Baseline,^†^ Mean (SD)  Mean (SD) at W12  Mean change (SD) at W12  P-value (change from baseline)  Percentage change from baseline W12 (95% CI) | n=220  76.7 (19.8)  82.5 (20.8)  5.8 (11.8)  <0.0001  9.71 (6.21, 13.21) | | n=316  75.2 (20.4)  80.3 (21.8)  5.1 (12.7)  <0.0001  8.39 (6.02, 10.76) | | n=253  74.9 (20.5)  79.8 (21.6)  4.9 (13.3)  <0.0001  8.66 (5.89, 11.43) | | n=150  73.7 (20.2)  78.7 (22.1)  5.0 (12.3)  <0.0001  8.43 (5.23, 11.62) | |
| **Maximum barefoot walking velocity** | | | | | | | | |
| Baseline,^†^ Mean (SD)  Mean (SD) at W12  Mean change (SD) at W12  P-value (change from baseline)  Percentage change from baseline at W12 (95% CI) | n=220  88.5 (26.9)  94.2 (26.4)  5.7 (14.5)  <0.0001  9.51 (5.66, 13.36) | | n=317  86.0 (26.7)  90.9 (27.4)  4.9 (15.7)  <0.0001  7.73 (5.21, 10.25) | | n=253  85.7 (26.1)  91.1 (27.9)  5.3 (16.3)  <0.0001  8.21 (5.30, 11.12) | | n=150  83.6 (26.4)  89.8 (28.5)  6.2 (15.2)  <0.0001  9.71 (5.96, 13.45) | |
| **Comfortable walking velocity with shoes** | | | | | | | | |
| Baseline,^†^ Mean (SD)  Mean (SD) at W12  Mean change (SD) at W12  P-value (change from baseline)  Percentage change from baseline at W12 (95% CI) | n=220  77.2 (19.3)  81.3 (18.6)  4.0 (12.1)  <0.0001  8.28 (4.24, 12.32) | | n=319  75.6 (20.2)  80.7 (20.9)  5.2 (13.1)  <0.0001  9.40 (6.44, 12.36) | | n=254  75.6 (20.2)  80.6 (21.3)  5.0 (12.4)  <0.0001  8.48 (5.99, 10.98) | | n=151  75.0 (20.3)  78.8 (22.1)  3.9 (12.6)  0.0002  7.18 (3.24, 11.13) | |
| **Maximum walking velocity with shoes** | | | | | | | | |
| Baseline,^†^ Mean (SD)  Mean (SD) at W12  Mean change (SD) at W12  P-value (change from baseline)  Percentage change from baseline at W12 (95% CI) | n=219  88.8 (24.1)  93.8 (24.3)  5.0 (12.2)  <0.0001  7.66 (4.71, 10.62) | | n=318  86.8 (25.3)  92.5 (27.0)  5.7 (15.7)  <0.0001  9.07 (6.17, 11.98) | | n=254  87.2 (25.0)  92.2 (27.3)  5.1 (16.2)  <0.0001  7.58 (4.80, 10.35) | | n=151  85.7 (25.7)  91.4 (28.7)  5.7 (15.7)  <0.0001  8.63 (4.80, 12.47) | |

^†^Baseline refers to baseline of the double-blind study, prior to first injection of the patients entering the cycle. CI, confidence interval; SD, standard deviation; W12, Week 12; WV, walking velocity.

**Supplementary Table 2** Mixed Model for Repeated Measures (MMRM) analyses of change in walking patterns from baseline to W12 across injection cycles in each of the four walking test conditions (aboBoNT-A doses combined)

|  | **Double-blind** | | **Open-label** | | | | | |
| --- | --- | --- | --- | --- | --- | --- | --- | --- |
| **Walking velocity (m/s)** | **First injection** | | **Second injection** | | **Third injection** | | **Fourth injection** | |
| **Comfortable barefoot WV (N=337)** | | | | | | | | |
| LS mean change from baseline^†^ (SE) at W12  95% CI for LS mean at W12 | 0.075 (0.01)  0.055, 0.096 | | 0.087 (0.009)  0.07, 0.104 | | 0.095 (0.01)  0.076, 0.114 | | 0.095 (0.012)  0.071, 0.120 | |
| **Maximum barefoot WV (N=337)** | | | | | | | | |
| LS mean change from baseline^†^ (SE) at W12  95% CI for LS mean at W12 | 0.077 (0.01)  0.057, 0.097 | | 0.088 (0.008)  0.072, 0.105 | | 0.101 (0.009)  0.082, 0.119 | | 0.102 (0.012)  0.078, 0.126 | |
| **Comfortable WV with shoes (N=338)** | | | | | | | | |
| LS mean change from baseline^†^ (SE) at W12  95% CI for LS mean at W12 | 0.058 (0.01)  0.038, 0.078 | | 0.072 (0.008)  0.056, 0.089 | | 0.083 (0.009)  0.064, 0.101 | | 0.080 (0.012)  0.055, 0.104 | |
| **Maximum WV with shoes (N=338)** | | | | | | | | |
| LS mean change from baseline^†^ (SE) at W12  95% CI for LS mean at W12 | 0.067 (0.01)  0.047, 0.087 | | 0.087 (0.009)  0.07, 0.104 | | 0.097 (0.01)  0.078, 0.115 | | 0.095 (0.012)  0.071, 0.119 | |
| **STEP LENGTH [m/step]** |  |  | |  | |  | |  |
| **Comfortable barefoot WV (N=337)** | | | | | | | | |
| LS mean change from baseline^†^ (SE) at W12  95% CI for LS mean at W12 | 0.024 (0.005)  0.013, 0.034 | | 0.031 (0.004)  0.023, 0.04 | | 0.039 (0.005)  0.029, 0.049 | | 0.038 (0.006)  0.026, 0.051 | |
| **Maximum barefoot WV (N=337)** | | | | | | | | |
| LS mean change from baseline^†^ (SE) at W12  95% CI for LS mean at W12 | 0.023 (0.005)  0.013, 0.033 | | 0.033 (0.004)  0.024, 0.041 | | 0.039 (0.005)  0.03, 0.049 | | 0.036 (0.006)  0.024, 0.048 | |
| **Comfortable WV with shoes (N=338)** | | | | | | | | |
| LS mean change from baseline^†^ (SE) at W12  95% CI for LS mean at W12 | 0.025 (0.005)  0.014, 0.035 | | 0.027 (0.004)  0.018, 0.035 | | 0.034 (0.005)  0.025, 0.044 | | 0.039 (0.006)  0.027, 0.051 | |
| **Maximum WV with shoes (N=338)** | | | | | | | | |
| LS mean change from baseline^†^ (SE) at W12  95% CI for LS mean at W12 | 0.023 (0.005)  0.012, 0.033 | | 0.029 (0.004)  0.021, 0.038 | | 0.040 (0.005)  0.031, 0.05 | | 0.034 (0.006)  0.021, 0.046 | |
| **CADENCE [steps/min]** |  |  | |  | |  | |  |
| **Comfortable barefoot WV (N=337)** | | | | | | | | |
| LS mean change from baseline^†^ (SE) at W12  95% CI for LS mean at W12 | 5.238 (0.927)  3.420, 7.056 | | 4.325 (0.780)  2.795, 5.854 | | 4.166 (0.871)  2.459, 5.873 | | 4.067 (1.129)  1.853, 6.280 | |
| **Maximum barefoot WV (N=337)** | | | | | | | | |
| LS mean change from baseline^†^ (SE) at W12  95% CI for LS mean at W12 | 6.668 (0.931)  4.844, 8.492 | | 5.539 (0.773)  4.025, 7.054 | | 5.972 (0.863)  4.279, 7.665 | | 6.529 (1.118)  4.338, 8.72 | |
| **Comfortable WV with shoes (N=338)** | | | | | | | | |
| LS mean change from baseline^†^ (SE) at W12  95% CI for LS mean at W12 | 3.405 (0.926)  1.589, 5.221 | | 4.232 (0.776)  2.711, 5.753 | | 4.110 (0.867)  2.411, 5.810 | | 2.835 (1.121)  0.636, 5.033 | |
| **Maximum WV with shoes (N=338)** | | | | | | | | |
| LS mean change from baseline^†^ (SE) at W12  95% CI for LS mean at W12 | 5.799 (0.934)  3.967, 7.632 | | 6.322 (0.774)  4.805, 7.839 | | 5.730 (0.865)  4.034, 7.426 | | 6.229 (1.116)  4.041, 8.417 | |

Data were analyzed using a MMRM to assess change from baseline to Week 12 of each cycle. This model includes the fixed categorical effects of test condition, visit, test condition-by-visit interaction, test condition-by-baseline interaction, and the baseline value as a fixed continuous covariate.
^†^Baseline refers to baseline of the double-blind study, prior to first injection of the patients entering the cycle. CI, confidence interval; LS, least squares; SE, standard error; W12, Week 12; WV, walking velocity.

**Supplementary Table 3.** WV by ambulatory category after the fourth injection (open-label Cycle 3) at Week 12

| **Ambulatory category^†^** | **Comfortable barefoot WV**  **N=150** | | | **Comfortable WV with shoes**  **N=151** | | | **Maximal barefoot WV**  **N=150** | | | **Maximal WV with shoes**  **N=151** | | |
| --- | --- | --- | --- | --- | --- | --- | --- | --- | --- | --- | --- | --- |
|  | **Baseline**^‡^ | **Fourth injection** | **Difference** | **Baseline**^‡^ | **Fourth injection** | **Difference** | **Baseline**^‡^ | **Fourth injection** | **Difference** | **Baseline**^‡^ | **Fourth injection** | **Difference** |
| **<0.4 m/s, n (%)** | 70 (47) | 57 (38) | -13 (-9) | 63 (42) | 49 (33) | -14 (-9) | 54 (36) | 43 (29) | -11 (-7) | 42 (28) | 36 (24) | -6 (-4) |
| **0.4–0.8 m/s, n (%)** | 80 (53) | 67 (45) | -13 (-9) | 74 (49) | 70 (46) | -4 (-3) | 60 (40) | 60 (40) | 0 (0) | 65 (43) | 57 (38) | -8 (-5) |
| **≥0.8 m/s, n (%)** | 0 | 26 (17) | 26 (17) | 14 (9) | 32 (21) | 18 (12) | 36 (24) | 47 (31) | 11 (7) | 44 (29) | 58 (38) | 14 (9) |

^†^Patients with a WV of at least 0.8 m/s are considered unlimited community ambulators, 0.4 to 0.8 m/s is considered community limited ambulators and patients with WV less than 0.4 m/s are considered household ambulators. ^‡^Baseline refers to baseline of the double-blind study, prior to first injection of the patients entering the cycle. N, total number of patients; n, number of patients; WV, walking velocity**.**

**Supplementary Table 4.** WV for patients in the ≥0.8 m/s ambulatory category^†^ at W12 after the fourth injection (open-label Cycle 3)

|  | **Comfortable barefoot WV**  **N=26** | **Comfortable WV with shoes**  **N=32** | **Maximal barefoot WV**  **N=47** | **Maximal WV with shoes**  **N=58** |
| --- | --- | --- | --- | --- |
| Baseline^‡^  Mean (SD)  Min, max | 0.69 (0.09)  0.38, 0.79 | 0.75 (0.13)  0.49, 1.00 | 0.91 (0.19)  0.55, 1.43 | 0.94 (0.24)  0.23, 1.67 |
| W12 after fourth injection  Mean (SD)  Min, max | 0.97 (0.12)  0.81, 1.17 | 0.98 (0.14)  0.81, 1.31 | 1.14 (0.24)  0.81, 2.00 | 1.14 (0.26)  0.81, 2.12 |
| Quantitative gain  Mean (SD)  Min, max | 0.27 (0.15)  0.04, 0.56 | 0.23 (0.16)  -0.02, 0.63 | 0.23 (0.22)  -0.25, 1.01 | 0.20 (0.24)  -0.40, 1.11 |

^†^Patients with a WV of at least 0.8 m/s are considered unlimited community ambulators. ^‡^Baseline refers to baseline of the double-blind study, prior to first injection of the patients entering the cycle. Q, quartile; SD, standard deviation; W12, Week 12; WV, walking velocity**.**
